# Analysis of popliteal artery location for high tibial and distal tuberosity osteotomy using contrast-enhanced computed tomography

**DOI:** 10.1186/s43019-022-00154-2

**Published:** 2022-05-08

**Authors:** Akiyoshi Mori, Takehiko Matsushita, Nobuaki Miyaji, Kanto Nagai, Daisuke Araki, Noriyuki Kanzaki, Tomoyuki Matsumoto, Takahiro Niikura, Yuichi Hoshino, Ryosuke Kuroda

**Affiliations:** Department of Orthopaedic Surgery, Kobe University Graduate School of Medicine, 7-5-1, Kusunoki-cho, Chuo-ku, Kobe, 650-0017 Japan

**Keywords:** High tibial osteotomy, Distal tuberosity osteotomy, Hybrid closed wedge, Popliteal artery, Safe angle

## Abstract

**Background:**

Our objective was to evaluate the location of popliteal artery (PA) in osteotomy planes during high tibial osteotomy (HTO) and to determine a safer angle for screw drilling to the tibial tuberosity during distal tuberosity osteotomy (DTO).

**Methods:**

Twenty knees in 20 patients who underwent contrast-enhanced computed tomography for cardiovascular diseases were examined. Osteotomy planes for open-wedge HTO (OWHTO) and hybrid closed-wedge HTO (hybrid CWHTO) were created using three-dimensional bone models. The distance from the posterior cortex of the tibia to the PA (dPC-PA) in the osteotomy planes was measured in the virtual osteotomy planes. The dangerous point (Point D1) was defined as the point 17.5 mm away from PA, setting the working length of the bone saw as 35 mm. The distance between the most medial point of the tibial cortex (Point M) and Point D1 in OWHTO and the most lateral point (Point L) and Point D1 in hybrid CWHTO were examined (dM-D1 and dL-D1, respectively). The location of Point D1 to the osteotomy line (%D1) was expressed as percentage, setting the start and end of the osteotomy line as 0% and 100%, respectively. To determine the safe angle for screw drilling in DTO, the angle between the line tangential to the medial cortex of the tibia and that passing through the center of the tibial tuberosity and PA were measured.

**Results:**

In OWHTO and hybrid CWHTO, the mean dPC-PA was 10.6 mm (6.9–16.5 mm) and 10.2 mm (7.3–15.4 mm), respectively. The mean dM-D1 in OWHTO was 25.9 mm (24.6–27.2 mm) and dL-D1 in hybrid CWHTO was 5.1 mm (2.9–7.4 mm). The mean %D1 was 47.6 ± 3.7% in OWHTO and 9.3 ± 4.1% in hybrid CWHTO, respectively. The minimal angle between the two lines in DTO was 35.2°.

**Conclusion:**

PAs could run within 10 mm from the posterior cortex in the osteotomy planes of HTO. Therefore, proper posterior protection is necessary when cutting posterior cortex. An angle of less than 35° against the medial cortex line would be safe for screw fixation to avoid vascular injury in DTO.

## Introduction

High tibial osteotomy (HTO) has been performed to treat patients with medial compartment osteoarthritis (OA) of the knee, with good clinical outcomes reported [1–5]. Over the past decades, medial open-wedge HTO (OWHTO) has gained popularity owing to its simple technique and relatively low complication rates [6]. Meanwhile, other new methods, including hybrid closed-wedge (hybrid CWHTO) and distal tuberosity osteotomies (DTO), are being increasingly performed [7–13].

Although HTO is an effective treatment for patients with medial knee OA, it is not free of complications. Numerous complications after HTO, including non-union, implant breakage, peroneal nerve palsy, infection, delayed wound healing, and neurovascular injuries, have been reported [6, 14, 15]. Among them, popliteal artery (PA) injury is a relatively rare complication with low prevalence rates ranging from 0.4% to 1.7% [15–17], but one of the most devastating ones. To prevent PA injury during HTO, several studies examined the location of the PA in osteotomy planes using computed tomography (CT) and magnetic resonance (MR) images [18–20]. Although these previous studies have provided useful information, the measurement method varies among the reports and potential problems exist in each method. In the studies using CT, the measurement setting of the osteotomy planes was not described in detail [18] or two-dimensional CT images were used [19], which may not be suitable for reproducing actual osteotomy planes during surgery. In addition, in the experimental model using MR images combined with contrast-enhanced CT, data from different subjects were mixed and the values could be different from the actual distances [20]. Furthermore, the risk of PA injury during cutting posterior cortex in HTO and screw drilling in DTO were not fully examined.

Therefore, the purpose of this study was to comprehensively evaluate the location of PA in the osteotomy planes for HTO and to determine the dangerous zone in HTO and screw drilling for fixation of the tibial tuberosity in DTO, using contrast-enhanced CT-based three-dimensional (3D) models.

## Materials and methods

### Patients

Contrast-enhanced CT images were obtained from 20 patients (from December 2012 to October 2018 at our institution; ten males, ten females; mean age 69.8 ± 15.3 years, mean height 164.2 ± 10.2 cm, mean weight 63.2 ± 8.4 kg) who underwent contrast-enhanced CT for acute cardiovascular diseases. Data from patients with inflammatory arthritis, retained hardware, and history of surgeries and fractures were excluded. This study was retrospectively conducted using the opt-out method according to the guidance of our hospital. Ethical approval was obtained from the institutional review board of our hospital.

### Image acquisition and processing

CT imaging using contrast-enhanced CT (Aquilion 64, Toshiba Medical Systems, Tokyo, Japan) was performed to examine the condition of the cardiovascular disease (100 kPv and 100 mA) with an image resolution of 0.4 mm per pixel and a slice thickness of 1 mm. Patients were placed in supine positions with both knees extended in neutral position, and the contrast medium (550 mg/kg) was injected through the vein in the forearm. CT images of 1-mm-thick slices were acquired in three different phases (pre-injection, arterial, and runoff phases), and images taken in the arterial phase were used. The data were transferred to compact discs, and 3D bone and vascular models were created using an image processing software 3-matic (Mimics Materialise, Leuven, Belgium).

### Creation of osteotomy planes and the minimum distance between the posterior cortex and the popliteal artery

**Fig. 1:**
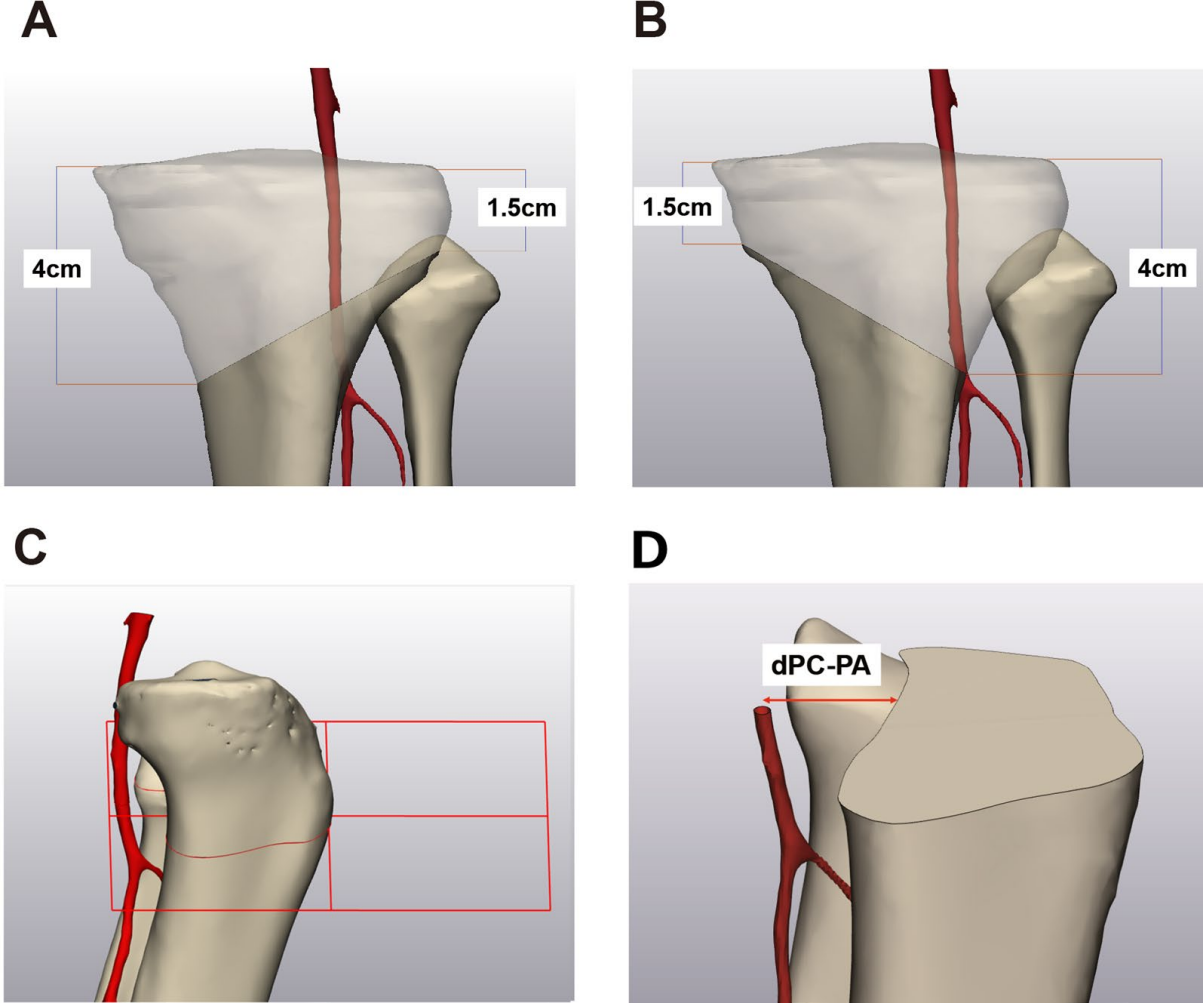
Osteotomy planes in three-dimensional bone models. **A** OWHTO. **B** Hybrid CWHTO. **C** Setting for the axial plane. The osteotomy plane was formed through the lines parallel to the tibial medial joint surface. **D** The distance between the posterior cortex and the popliteal artery (dPC-PA) was measured on the axial plane

First, a coronal plane was built through the tips of the medial and lateral malleoli and the fibula head in the 3D bone model. Next, to create transverse osteotomy planes for OWHTO, a line from a point 4 cm distal to the medial joint surface to a point 1.5 cm distal to the lateral joint surface, which corresponded to the fibular head level, was drawn on the coronal plane. Then, an axial plane was formed through the lines parallel to the tibial medial joint surface (posterior slope). For hybrid CWHTO, a line was drawn from a point 4 cm distal to the lateral joint surface to a point 1.5 cm distal to the medial joint surface on the coronal plane. Then an axial plane was formed through the lines parallel to the tibial medial joint surface on the lateral view (Fig. 1). The minimum distance between the posterior cortex and the PA (dPC-PA) was measured on the axial plane.

### Dangerous points during high tibial osteotomy

According to the previous study, the working length of the saw bone was set as 35 mm and the dangerous distance from the center of the saw bone to PA was set as 17.5 mm (half length of the working distance) [19]. The dangerous point (Point D) was defined as the point where the posterior edge of the bone saw can hit PA if the center of the saw is positioned at that point (the point 17.5 mm away from the most anterior wall of PA). The location of Point D was examined in two different cutting directions (parallel cutting: cutting parallel to the ground; oblique cutting: cutting obliquely from the most medial point to posterior direction).

**Fig. 2:**
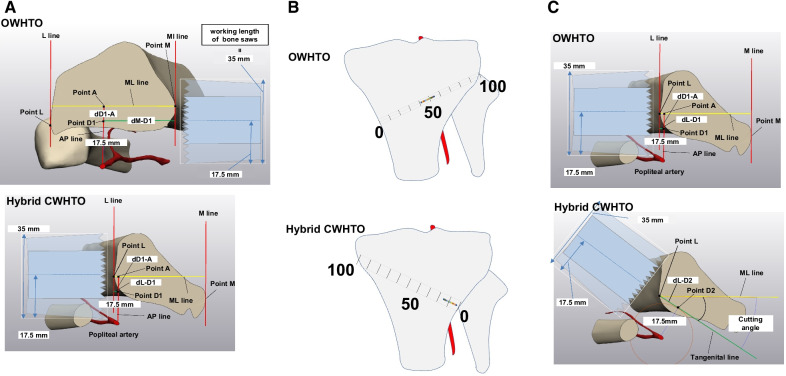
**A** Parallel cutting. Point M: the most medial Point M line; a line that was perpendicular to the ground and passed Point M. L line: a line that was perpendicular to the ground and most lateral edge of the tibia. Line ML: a line drawn from Point M in parallel to the ground until reaching L line. AP line: drawn from the most anterior part of PA until reaching the point on ML line. Point A: the crossing point of AP line and ML line. Point D1: the point located 17.5 mm away from the most anterior part of popliteal artery on AP line. dM-D1: the distance between Point M and Point D1. dD1-A: the distance between Point D1 and Point A. dL-D1: the distance between Point L and Point D1. dL-D2: the distance between Point L and Point D2. **B** Relative location of D1 in the osteotomy line. %D1: the ratio of dM-D1 (green line) or dL-D1 to the length of ML line (yellow line). The starting point of the osteotomy was defined as 0%, and the end of the osteotomy line was defined as 100%. The lower illustration shows the distribution of location of popliteal artery based on %D1 in OWHTO and hybrid CWHTO. **C** Oblique cutting. A circle centering the most anterior point of popliteal artery with a radius of 17.5 mm was drawn, and a tangential line to the circle was drawn from Point M. The contact point was defined as Point D2. Cutting angle: the angle between ML line and the tangential line. dM-D2: the distance between Point M and Point D2

1. Parallel cutting
  Lines that were perpendicular to the ground and pass the most medial point (Point M) and the most lateral point of the tibia in the osteotomy plane were drawn (M line and L line, respectively). Then, a line perpendicular to the M line was drawn from Point M in parallel to the ground until reaching the L line (ML line). A perpendicular line to the ML line was drawn from the most anterior part of PA until reaching the point (Point A) on the ML line (AP line). The point located 17.5 mm away from the most anterior part of PA on the AP line was defined as Point D1. In OWHTO, the distance between the M line and Point D1 (dM-D1) was measured as the saw length reaching the Point D1. The distance between Point D1 and the ML line (dD1-A) was also measured to determine the location of the center of the saw relative to the ML line (Fig. 2A). In hybrid CWHTO, the distance between Point L and the Point D1 (dL-D1) was measured (Fig. 2A).
  To estimate the location of PA during surgery, the locations of D1 in OWHTO and hybrid CWHTO were expressed as percentage of the length of the osteotomy line (%D1) using the formula: (dM-D1/length of ML line) × 100 and (dL-D1/length ML line) × 100, respectively. The starting point of the osteotomy was defined as 0%, and the end of the osteotomy line was defined as 100% (Fig. 2B).
2. Oblique cutting
  A circle centering the most anterior point of PA with a radius of 17.5 mm was drawn. Next, a tangential line to the circle was drawn from Point M, and the contact point was defined as Point D2 in OWHTO. The angle forming the ML line and the tangential line was measured as cutting angle. The distance between Point M and Point D2 (dM-D2) was also measured (Fig. 2C). In hybrid CWHTO, the tangential line was drawn from Point L and the distance between the Pint L and Point D2 (dL-D2) was measured (Fig. 2C).

### Safe angle for screw drilling to the tibial tuberosity in DTO

**Fig. 3:**
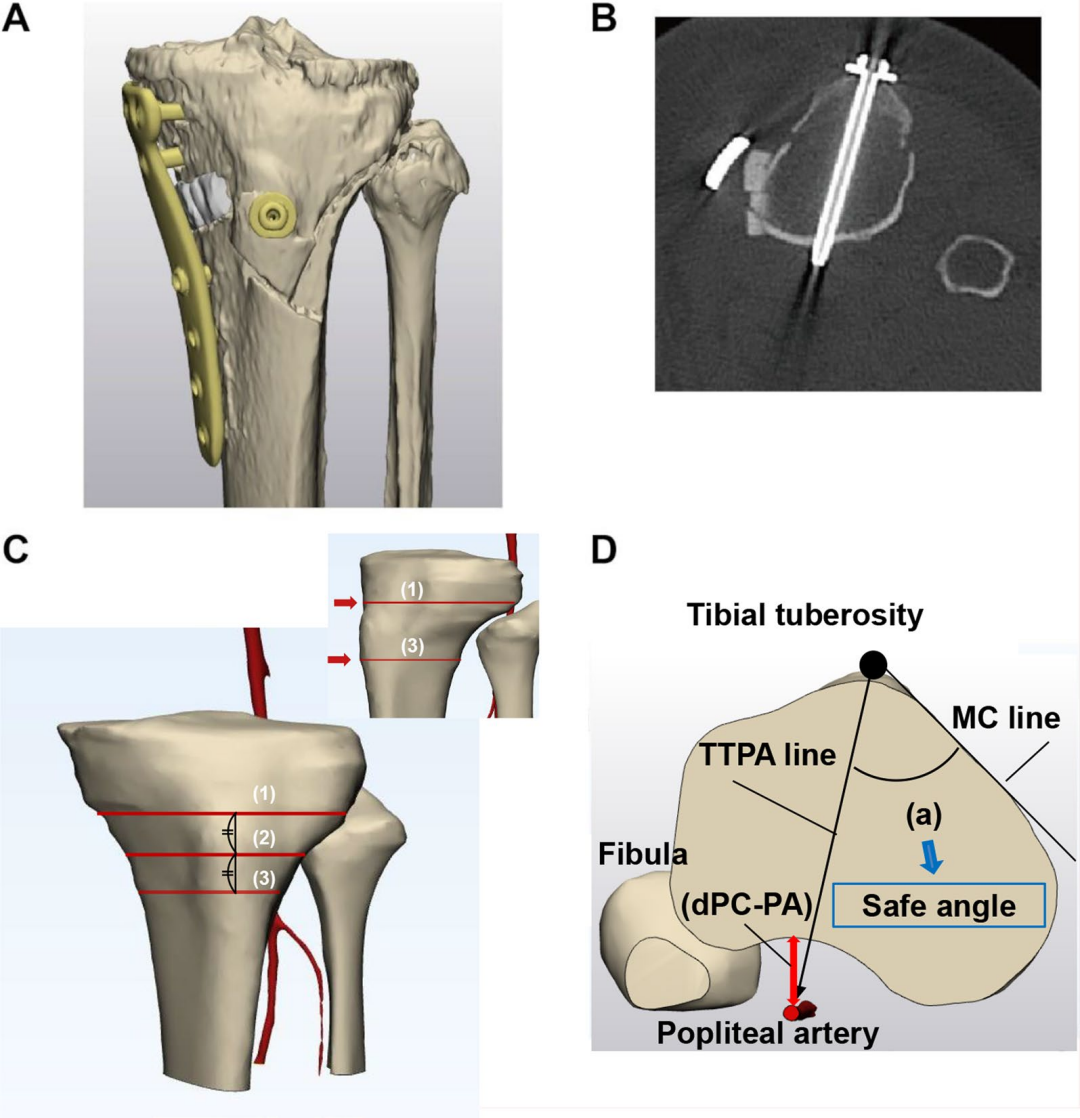
**A** Three-dimensional bone models after DTO. **B** An axial plane in the CT image after DTO: an anteroposterior screw for bicortical fixation of the tibial tuberosity in DTO. **C** Most proximal point (1), midpoint (2), and most distal point (3) in the tibial tuberosity. **D** An axial plane. (a): the angle between medial cortex (MC) line and tibial tuberosity and popliteal artery (TTPA) line. dPC-PA: the distance between the posterior cortex and the popliteal artery

To determine the safe angle for screw drilling to the tibial tuberosity in DTO, medial cortex of the tibia was set as reference. Axial planes were made at three different levels through the points on the tibial tuberosity: the most proximal point, midpoint, and the most distal point (Fig. 3). The most proximal and distal points were arbitrarily chosen on the basis of the morphology of the tuberosity in the 3D images (Fig. 3C). Then the angle between the line tangential to the medial cortex of tibia (MC line) and the line connecting the center of the tibial tuberosity and PA (TTPA line) was measured to determine the screw angle directing toward the PA (Fig. 3D).

### Statistical analysis

The sample size calculation showed that, if the standard deviation of dPC-PA was estimated as 2.5 mm on the basis of previous reports [19, 20], the minimal sample size would be 17 to set 95% of confidence intervals of 2.4 mm (EZR version 1.55, Jichi Medical University, Tokyo, Japan) [21]. A priori power analysis using G*Power 3.1 (Christian Albrecht University, Kiel, Germany) [22] indicated that a sample size of at least 15 patients per group for the comparison between OWHTO and hybrid CWHTO, and at least 16 patients per group for comparison among the three levels, was necessary to detect an intergroup difference in each parameter with an alpha of 0.05 and a power of 80%, based on pilot testing data. Student’s *t*-test was used to compare the difference between OWHTO and hybrid CWHTO. One-way analysis of variance was used to compare the differences among the three levels in DTO. For all analyses, statistical significance was set at *p* < 0.05. Data were reported as mean ± standard deviation or mean (range, maximum–minimum).

## Results

### The minimum distance between the posterior cortex and the popliteal artery

In the osteotomy planes of OWHTO and hybrid CWHTO, the mean dPC-PA was 10.8 ± 2.4 mm (range 6.9–16.5 mm) and 10.3 ± 2.2 mm (7.3–15.4 mm), respectively. There was no statistically significant difference between OWHTO and hybrid CWHTO (Table 1).

**Table 1:** The distance between the posterior cortex of the tibia and the popliteal artery in the osteotomy planes for open-wedge high tibial osteotomy and hybrid closed-wedge high tibial wedge osteotomy

	OWHTO	Hybrid CWHTO	Statistics
dPC-PA (mm)	10.8 ± 2.4 (6.9–16.5)	10.3 ± 2.2 (7.3–15.4)	n.s.

*OWHTO* open-wedge high tibial osteotomy, *CWHTO* closed-wedge high tibial osteotomy, *dPC-PA* distance between the posterior cortex of the tibia and the popliteal artery. Data are shown as mean ± standard deviation (range). *n.s.* no statistically significant difference

### Dangerous points during high tibial osteotomy

In the parallel cutting for OWHTO, the mean dM-D1 and dD1-A were 25.9 ± 2.9 mm and 6.0 ± 2.0 mm, respectively. In hybrid CWHTO, the mean dL-D1 and dD1-A were 5.1 ± 1.7 mm and 5.6 ± 2.1 mm, respectively. In the oblique cutting for OWHTO, the mean dM-D2 was 31.1 ± 3.1 mm, and the cutting angle was 11.2 ± 2.3°. In hybrid CWHTO, the mean dL-D2 was 12.7 ± 2.2 mm, and the cutting angle was 31.8 ± 5.6°. The mean %D1 was 47.6% ± 3.7% and 9.3% ± 4.1% in OWHTO and hybrid CWHTO, respectively (Table 2) (Fig. 2B).

**Table 2:** Results of the analysis of dangerous points in the osteotomy planes

	OWHTO	Hybrid CWHTO
Parallel cutting		
dM-D1/dL-D1 (mm)	25.9 ± 2.9 (24.6–27.2)	5.1 ± 1.7 (2.9–7.4)
dD1-A (mm)	6.0 ± 2.0 (3.3–8.9)	5.6 ± 2.1 (2.9–8.6)
%D1 (%)	47.8 ± 3.8 (41.7–54.2)	9.3 ± 4.0 (2.6–15.1)
Oblique cutting		
dM-D2 (mm)	31.1 ± 3.1 (27.1–35.2)	12.7 ± 2.2 (9.9–15.8)
dL-D2 (mm)	11.2 ± 2.3 (8.2–15.3)	31.8 ± 5.6 (20.1–46.2)
Cutting angle (°)	11.2 ± 2.3 (8.2–15.3)	31.8 ± 5.6 (20.1–46.2)

*OWHTO* open-wedge high tibial osteotomy, *CWHTO* closed-wedge high tibial osteotomy, *dM-D1* distance between Point M and Point D1 in OWHTO, *dL-D1* distance between Point L and Point D1 in hybrid CWHTO, *dD1-A* distance between Point D1 and Point A, *%D1* relative location of Point D1 in the osteotomy line, *dM-D2* distance between Point M and Point D2, *cutting angle* angle between ML line and tangential line. Data are shown as mean ± standard deviation (range). *N* = 20

### Safe angle for screw drilling to the tibial tuberosity in DTO

The mean dPC-PAs in the planes at the tibial tuberosity are presented in Table 3. There was no statistically significant difference among the three levels.

**Table 3:** The distance between the posterior cortex of the tibia and the popliteal artery in the planes at the three levels of distal tuberosity

Level of tibial tuberosity	dPC-PA (mm)
Most proximal level	12.7 ± 2.6 (8.9–16.7)
Mid-level	13.1 ± 2.1 (9.2–16.2)
Most distal level	13.2 ± 2.0 (8.2–15.7)
Statistics	n.s.

*dPC-PA* distance between the posterior cortex of the tibia and the popliteal artery

Data are shown as mean ± standard deviation (range). *n.s.* no statistically significant difference

The average angles between the MC line and the TTPA line at the proximal, mid, and distal levels were 47.8 ± 4.2° (40.2–54.0°), 45.2 ± 5.3° (35.2–50.2°), and 45.8 ± 5.8° (37.9–52.4°), respectively. There was no statistically significant difference among the three levels (Table 4). The minimum angle in all the measured planes at the three levels was 35.2° at the mid-level.

**Table 4:** The average angles between the MC line and the TTPA line at the proximal, mid, and distal levels

Level of tibial tuberosity	Angle between MC line and TTPA line (°)
Most proximal level	47.8 ± 4.2 (40.2–54.0)
Mid-level	45.2 ± 5.3 (35.2–52.2)
Most distal level	45.8 ± 5.8 (37.9–52.4)
Statistics	n.s.

*MC line* medial cortex line, *TTPA line* tibial tuberosity–popliteal artery line

Data are expressed as mean ± standard deviation (range). *n.s.* no statistically significant difference

## Discussions

The present study showed three main findings. First, PAs run approximately 7–16 mm away from the posterior cortex in the osteotomy planes for both OWHTO and hybrid CWHTO without significant differences. Second, PA locates approximately at 42–54% and 3–15% of the osteotomy line in OWHTO and hybrid CWHTO, respectively. Third, the minimum screw drilling angle from the tibial tuberosity toward the PA, with the medial tibial cortex as reference, was 35.2°, suggesting that the safe angle in DTO was less than 35° to the MC line.

Previously, several studies examined the distance between PA and the posterior cortex in the osteotomy planes for HTO. Bisicchia et al. examined the distance between the PA and the posterior cortex in cadavers using contrast-enhanced CT images and reported that the mean distance was 9.6 ± 2.1 mm in the osteotomy planes for OWHTO [18]. Kang et al. examined the distance between the PA and the posterior cortex in the virtual osteotomy planes of OWHTO using contrast-enhanced CT in patients with peripheral artery diseases and reported a value of 15 mm with a 99% confidence interval of 9–21 mm [19]. Choi et al. reported that the mean distance measured from MR images was 13.0 ± 2.0 mm in osteotomy planes of OWHTO [20]. In this study, the mean minimal distance between the posterior cortex and PA in the osteotomy planes for OWHTO was 10.8 ± 2.5 mm (Table 5). Although similar mean values of dPC-PA were reported from previous studies, there were differences in the mean distance between posterior cortex and PA among the previous studies. The difference could be partly due to methodological difference among the studies. In the study using cadavers, the distance may have changed during dissection. In addition, it would have been difficult to consistently determine the closest point from PA. While similar measurement methods to Kan’s report were used in this study, approximately 5 mm difference in average dPC-PA was observed. One reason could be the difference in osteotomy plane. In our study, the osteotomy plane was created to include the point 4 cm distal to the medial joint line and the point 1.5 cm distal to the lateral joint level. In addition, the sagittal plane was adjusted by the tibial joint surface. In Kang’s study, the osteotomy plane was reconstructed at 3.5 cm below the joint line and perpendicular to the tibial axis. Therefore, the difference in osteotomy plane may have caused the measurement difference. In the report by Choi, dPC-PA was measured in the experimental models using MR images combined with contrast-enhanced CT from different subjects. Therefore, the values may be different from the actual distances. Together with our data, these reports suggest that PAs run approximately 10 mm away from the posterior cortex in the osteotomy planes for OWHTO. Further, the distance was also measured in the planes for hybrid CWHTO in the present study. The mean distance was 10.3 ± 2.2 mm in hybrid CWHTO, and there was no significant difference between the planes for OWHTO and hybrid CWHTO. Therefore, PAs run approximately 10 mm away from the posterior cortex at proximal tibia levels, and the risk of PA injury was similar regardless of the osteotomy technique. Since it is very difficult to avoid the PA just by controlling the bone saw during osteotomy and the distance can be even shorter in some patients, careful attention and adequate protection are essential to prevent PA injuries during HTO.

**Table 5:** Previous reports and this study on the distance between the posterior cortex and the popliteal artery in the osteotomy plane

Author (year)	Method (number of subjects)	Osteotomy technique	Distance between posterior cortex and popliteal artery
Bisicchia et al. (2015)	Contrast-enhanced CT (six cadavers)	OWHTO	9.6 ± 2.1 mm
Kang et al. (2020)	Contrast-enhanced CT (45 patients)	OWHTO	15 mm (99% CI 9–21)
Choi et al. (2019)	MRI–CT matching model MRI (16 patients) Contrast-enhanced CT (52 subjects)	OWHTO	13.0 ± 2.0 mm
This study	Contrast-enhanced CT (20 patients)	OWHTO Hybrid CWHTO	10.8 ± 2.5 mm 10.3 ± 2.3 mm

*OWHTO* open-wedge high tibial osteotomy, *Hybrid CWHTO* hybrid closed-wedge high tibial osteotomy, *CT* computed tomography, *MRI* magnetic resonance imaging

Kang et al. reported that the working width of the saw oscillates reached up to 35 mm [19]. Notably, the minimal distance in our study was approximately 7 mm. Taking the working length of the bone saw into consideration, the dangerous point based on the working length of the bone saw was also examined. The mean distance to the dangerous point from the most medial point in OWHTO was 25.9 ± 2.9 mm, which corresponds to 47.6 ± 3.7% of the osteotomy line, when the center of the saw was positioned 6.0 ± 2.0 mm posteriorly. The results suggest that, if osteotomy starts from the position approximately 5–6 mm posterior to the most medial point and progresses in parallel to the ground, the tip of the saw will come into contact with PA. Therefore, surgeons should exercise great caution when cutting the bone close to this point. Meanwhile, in hybrid CWHTO, PA was located within close proximity to the starting point of osteotomy. Therefore, surgeons should cut the posterior cortex while retracting PA posteriorly.

This study used CT images taken in the knee joint extension position. Several reports have examined the effect of knee flexion angle on the distance between the PA and the posterior cortical bone of the tibia. Kim et al. reported that, between 0° and 90° flexion, the PA was farthest away from the posterior tibia at 90° flexion with a mean of 11 mm at 2.0 cm below the joint line [23]. Meanwhile, Choi et al. reported that the distance increased by only 1.3–1.7 mm at 90° flexion compared with 0° flexion in the virtual osteotomy planes [20]. Shetty et al. reported that, in 85% of the cases, the PA receded from the posterior tibia in knee flexion, whereas in the remaining cases, it approached the posterior tibia [24]. Thus, there is no clear consensus. In any case, the PA may not move to a position where a bone chisel or bone saw cannot reach it by knee flexion alone. Although the artery is movable, its mobility is not likely to help prevent damage to the arterial wall once the bone saw hits PA. Meanwhile, when chisels contact with PA, there may be a chance that PA moves aside posteriorly without being damaged. However, as this is not guaranteed, correct placement of the retractor just below the posterior cortical bone of the tibia is a sure way to prevent damage to PA.

Recently, DTO has attracted the attention of surgeons considering previous reports on the deterioration of patellofemoral joints after OWHTO [25–27]. In DTO, screw fixation for the tibial tuberosity is generally required. Meanwhile, case reports on PA injuries by tibial tuberosity fixation during tibial tuberosity transfer have been reported [28, 29]. Hernigou et al. previously examined the safe zone and danger zone for screw drilling during tibial tuberosity transfer and reported that the direction to the medial one-third of the tibia was the safest zone and the lateral upper side of tibia was the most dangerous zone [30]. Yang et al. also examined the safe zone for surgeries on proximal tibia using MR images. They reported that neurovascular structures were observed lateral to the posterior middle line of the tibia and suggested that the safe zone was the medial half of the tibia if penetration of the posterior cortex was needed during surgeries [31]. Furthermore, they measured the angle between the line passing through the most anterior part of the tibial tuberosity to the PA and the AP axis, and the angle was < 10°. However, those references including the AP axis could be affected by the rotation of the lower leg, and the estimation of the safe direction may not be fully reliable. In the present study, the safe angle for screw drilling was determined in relation to the medial cortex line since the medial cortex line can be easily identified during surgery and the effects of rotation can be avoided. Considering that the minimum angle in our study was 35.2°, it was suggested that an angle of less than 35° against MC line would be safe for screw drilling in DTO.

This study has several limitations. First, since the PAs were identified using contrast-enhanced CT, the vascular wall was not considered. Therefore, the distance from the posterior cortex to the PA, when including the vascular wall, will be shorter than our measured dPC-PA. Second, although the minimal distance between the posterior cortex and PA in our patients was 7 mm, the distance can be shorter than 7 mm in other patients. Third, during DTO, the distal part shifts laterally after opening the gap. Therefore, the vascular course changes, and consequently the distance and safe angle may change. However, the lateral shift of the distal part will shift the neurovascular structure laterally, and the safe angle is not likely to be less than 35°. Fourth, the subjects of this study were 20 patients with cardiovascular disease who underwent contrast-enhanced CT for cardiovascular diseases, not patients with knee OA with altered alignment who are eligible for HTO surgeries. However, Lee et al. reported that, in their MRI study, the location of PA distal to the joint level was not significantly different between the arthritic and non-arthritic groups [32]. Fifth, this study evaluated only CT images taken in the knee joint extension position. Since the distance between PA and the posterior cortical bone can change depending on the knee flexion angle, the values in our study may not be utilized if a surgeon uses a different knee flexion angle during surgery.

Despite the limitations, we believe that the present study provides useful information for preventing PA injury during HTO and DTO.

## Conclusion

As PA may locate within 10 mm of the posterior tibial cortex in the osteotomy planes of HTO, proper posterior protection is necessary regardless of the osteotomy technique. An angle of less than 35° against the medial cortex line would be safe for screw fixation in DTO.

## Data Availability

The data and materials that support the findings of this study are available upon request.

## Abbreviations

PA: Popliteal artery
HTO: High tibial osteotomy
DTO: Distal tuberosity osteotomy
CT: Computed tomography
OWHTO: Open-wedge HTO
CWHTO: Closed-wedge HTO
dPC-PA: Distance from the posterior cortex of the tibia to the PA
OA: Osteoarthritis
AP: Anteroposterior
MR: Magnetic resonance
3D: Three-dimensional
dM-D1: Distance between Point M and Point D1
dL-D1: Distance between Point L and Point D1
%D1: Ratio of length of distance between Point M and Point L to length of dM-D1
dD1-A: Distance between Point D1 and Point A
ML line: Line tangential to the medial lateral of tibia
dM-D2: Distance between Point M and Point D2
dL-D2: Distance between Point L and Point D2
MC line: Line tangential to the medial cortex of tibia
TTPA line: Line connecting the center of the tibial tuberosity and PA
